# EEG-derived brain graphs are reliable measures for exploring exercise-induced changes in brain networks

**DOI:** 10.1038/s41598-021-00371-x

**Published:** 2021-10-21

**Authors:** Daniel Büchel, Tim Lehmann, Øyvind Sandbakk, Jochen Baumeister

**Affiliations:** 1 Department Sport & Health, Exercise Science & Neuroscience Unit, Paderborn University, Warburger Str. 100, 33098 Paderborn, Germany; 2 Department of Neuromedicine and Movement Science, Centre for Elite Sports Research, Norwegian University of Science and Technology, Trondheim, Norway

**Keywords:** Neural circuits, Decision

## Abstract

The interaction of acute exercise and the central nervous system evokes increasing interest in interdisciplinary research fields of neuroscience. Novel approaches allow to monitor large-scale brain networks from mobile electroencephalography (EEG) applying graph theory, but it is yet uncertain whether brain graphs extracted after exercise are reliable. We therefore aimed to investigate brain graph reliability extracted from resting state EEG data before and after submaximal exercise twice within one week in male participants. To obtain graph measures, we extracted global small-world-index (SWI), clustering coefficient (CC) and characteristic path length (PL) based on weighted phase leg index (wPLI) and spectral coherence (Coh) calculation. For reliability analysis, Intraclass-Correlation-Coefficient (ICC) and Coefficient of Variation (CoV) were computed for graph measures before (REST) and after POST) exercise. Overall results revealed poor to excellent measures at PRE and good to excellent ICCs at POST in the theta, alpha-1 and alpha-2, beta-1 and beta-2 frequency band. Based on bootstrap-analysis, a positive effect of exercise on reliability of wPLI based measures was observed, while exercise induced a negative effect on reliability of Coh-based graph measures. Findings indicate that brain graphs are a reliable tool to analyze brain networks in exercise contexts, which might be related to the neuroregulating effect of exercise inducing functional connections within the connectome. Relative and absolute reliability demonstrated good to excellent reliability after exercise. Chosen graph measures may not only allow analysis of acute, but also longitudinal studies in exercise-scientific contexts.

## Introduction

While the interaction of acute endurance exercise and the cardiovascular system is well understood, research in the last decade started to rather unfold the effect of acute and long-term exercise on the central nervous system^1–3^. Thus, growing evidence suggests that metabolic changes induced by exercise are associated with systematic modulations of brain function due to neurochemical changes within the central nervous system^2^. Several neuroimaging studies have shown that these acute changes induce modulations of brain function that persist at rest after exercise cessation^1^. Hereof, the use of electroencephalography (EEG) appears feasible for field-application due to its fast applicability, high degree of mobility and excellent temporal resolution^4^. Nevertheless, EEG data obtained during high intensity exercise is massively contaminated by artefacts induced by muscle and movement artefacts^5^. Therefore, the EEG resting state after exercise provides a valuable insight into short-term exercise-induced modulations of brain function^1^. In particular, EEG resting state data revealed systematic exercise-induced regional phenomena like altered power spectral density^3^, alpha peak frequency^6^ or oscillatory microstate patterns^7^ across participants. However, while the abovementioned findings focused on changes of locally segregated activity of neural patches, treating the brain as a dense connectome integrating locally segregated neural structures evokes a new and interesting perspective in exercise neuroscience^8–10^.

For analyzing brain networks, graph theory offers a promising opportunity to model pairwise communications between the elements of large-scale brain networks to abstract neurophysiological parameters. Brain graphs treat single sensors or neural patches as network nodes and the connections between those nodes as edges^11^ and can therefore express mathematical characteristics of dense, multivariate connectomes^12^. For instance, measures like the clustering coefficient (CC), the characteristic path length (PL) or the small-world-index (SWI) of a network help to abstract key characteristics of brain networks where the aspects of local segregation and global integration are combined to describe brain network efficiency^12–14^. Although brain network analysis has mainly been applied in clinical settings to distinguish brain function between special and healthy populations^13,15,16^, first investigations applied graph theory in exercise science contexts. Recent findings observed that acute aerobic exercise modulates the connectome in an intensity-dependent fashion, since global efficiency increased more at moderate in comparison to lower intensity cycling^8^. Moreover, a loss of network efficiency, as indicated by a reduction of the frontal clustering coefficient, was reported after exhaustive exercise^17^ and an exhaustive motor-cognitive dual-task protocol inducing both physiological as well as cognitive exhaustion^9^. Taken together, these findings suggest that brain graph reveal a promising tool to investigate the modulatory effect of acute and long-term exercise on the connectome. Nevertheless, especially for intra-individual analysis of brain networks metrics, a reliable extraction of graph outcomes^18^ seems paramount.

To date, investigations of reliability solely focused on relative reliability, in which fair to good intraclass-correlation-coefficients (ICCs) were reported for graph measures computed from resting state EEG data in sitting position in sound-attenuated rooms^18–20^. The moderating effects on reliability were assigned to aspects related to graph extraction like the measure of functional connectivity (FC) estimation, the frequency band of interest or the number of epochs analyzed^18–20^. Additionally, it was observed that the topographical level of graph measures affects brain graph reliability, as global graphs released more reliable metrics compared to regional or local graphs^19^. Altogether, graph measures from resting EEG seem to provide a reliable insight into brain function when key parameters of graph extraction are considered.

However, for a longitudinal application of graphs in exercise-related settings, it needs to be clarified whether graph outcomes assessed after exercise are reliable either. As reported by Shen et al., the architecture of brain networks highly depends on consistent structural connections, but is further modulated by dynamics of functional pathways. Accordingly, neuroimaging studies suggest that functional task-related brain networks are more reliable than unconstrained resting state networks^21,22^. Since acute bouts of exercise modulate the cortical activity and induce the activations of functional pathways that persist after exercise^1,2^, a resting assessment of brain network post exercise may differ from an unconstrained resting state before exercise. Therefore, we expect that not only the degree of connectivity^8–10^, but also the reliability of this connections might be modulated by exercise-induced systematic changes in brainfunction. So far, no study has investigated the reliability of brain network metrics explicitly after an acute bout of exercise.

Therefore, the present study aimed to investigate test–retest reliability of brain graphs derived from both (i) an unconstrained resting state before exercise as well as (ii) a resting state following an acute bout of low intensity endurance exercise while running on a treadmill in male participants. We hypothesized that exercise-induced functional pathways^2^ result in a modulation of test–retest reliability of graph measures immediately after exercise compared to the unrestricted resting state. Since the FC estimator has been shown to modulate brain network reliability^19^, we aimed to observe the effect of preceding exercise on brain graph reliability for two different FC estimates of interest (wPLI vs. Coh). The findings of the present study may help to better understand whether brain graphs are reliable measures to assess exercise-induced modulations of large-scale brain networks in repeated measures designs.

## Results

### Physiological outcomes

An overview of physiological outcomes including mean ± SD and ICC estimations is presented in Table 1. Analysis of physiological data revealed systematic changes in physiological responses to exercise between session 1 and session 2. We observed an interaction effect indicating that Lac_post_ was lower at Session II compared to Session I (p = 0.01) and a main-effect demonstrating that BS reduced from session I to session II (p < 0.01). Further, BS and HR_rest_ revealed significant main-effects of exercise, with higher values at POST compared to PRE (p < 0.05).

**Table 1:** Overview of results revealed from reliability analysis for physiological outcomes before and after 10 min of running on the treadmill.

	Descriptives		Relative reliability		Absolute reliability	
	Session 1	Session 2	ICC	[LB UB]	CoV (%)	[LB UB]
Lac_PRE_ (in mmol/l)	1.0 ± 0.4	1.0 ± 0.3	0.67	[0.26 0.87]	22.64	[31.96 14.69]
Lac_POST_ (in mmol/l)	1.4 ± 0.7	1.2 ± 0.5	0.84	[0.34 0.95]	18.93	[33.17 11.15]
BS_PRE_	7.3 ± 1.6	7.0 ± 1.2	0.34	[0.0 0.72]	15.60	[20.94 10.45]
BS_POST_	11.1 ± 1.8	9.8 ± 1.8	0.65	[0.0 0.89]	10.02	[17.17 5.70]
HR_rest PRE_ (%HR_max_)	32.5 ± 11.6	32.3 ± 7.53	0.86	[0.62 0.95]	7.68	[11.99 4.74]
HR_rest POST_ (%HR_max_)	39.2 ± 7.4	38.0 ± 6.64	0.76	[0.43 0.91]	8.67	[13.47 5.36]
HR_run_ (%HR_max_)	68.0 ± 5.2	65.3 ± 6.1	0.58	[0.13 0.83]	5.49	[7.87 3.45]

Data are presented as outcome means ± standard deviations, Intraclass-Correlation-Coefficients (ICC) and Coefficient of Variation (CoV) including lower (LB) and upper bound (UB) of the 95% confidence interval of two sessions performed within 1 week and statistical outcomes of repeated measures ANOVA with the factors Exercise (PRE vs. POST) and Session (Session I vs. Session II) performed at p < 0.05. Blood lactate values (Lac) obtained from the right earlobe and rate of perceived exertion (RPE) were assessed before (PRE) and immediately after (POST) running on the treadmill. Average resting heart rate (HR _rest_) is determined as the 5-min average heart rate (HR) recorded in a sitting position, HR in running (HR_run_) is obtained as the average HR during 10-min of running at 50% of the individual VO_2peak_. HR measures are presented as the percentage of the individual HR_max_.

ICC analysis revealed good to excellent correlation values for Lac (PRE: 0.66 vs. POST: 0.84) and HR_rest_ (PRE: 0.83 vs POST: 0.83) while ICC for BS increased from REST to POST (PRE: 0.34 vs. POST: 0.65).

### Graph analysis

ANOVA on EEG-derived graph measures revealed significant main-effects on Coh-based graph measures, where SWI increased from PRE to POST for both sessions in the alpha-2 band (p = 0.006, F = 10.35) and decreased in the beta-1 (p = 0.003, F = 12.48) and beta-2 bands (p = 0.002, F = 15.48). In line with these changes, Coh-based CC increased in the alpha-1 (p = 0.02, F = 6.56) and alpha-2 band (p = 0.002, F = 14.49), while CC decreased in the beta-1 (p = 0.02, F = 6.61) and beta-2 bands (p = 0.01, F = 9.01) from PRE to POST in both sessions. Furthermore, PL decreased in the alpha-1 (p = 0.04, F = 5.19) and alpha-2 bands (p = 0.0006, F = 10.06) and decreased in the beta-1 (p = 0.01, F = 8.56) and beta-2 bands (p = 0.005, F = 15.47) from PRE to POST in both sessions.

**Figure 1:**
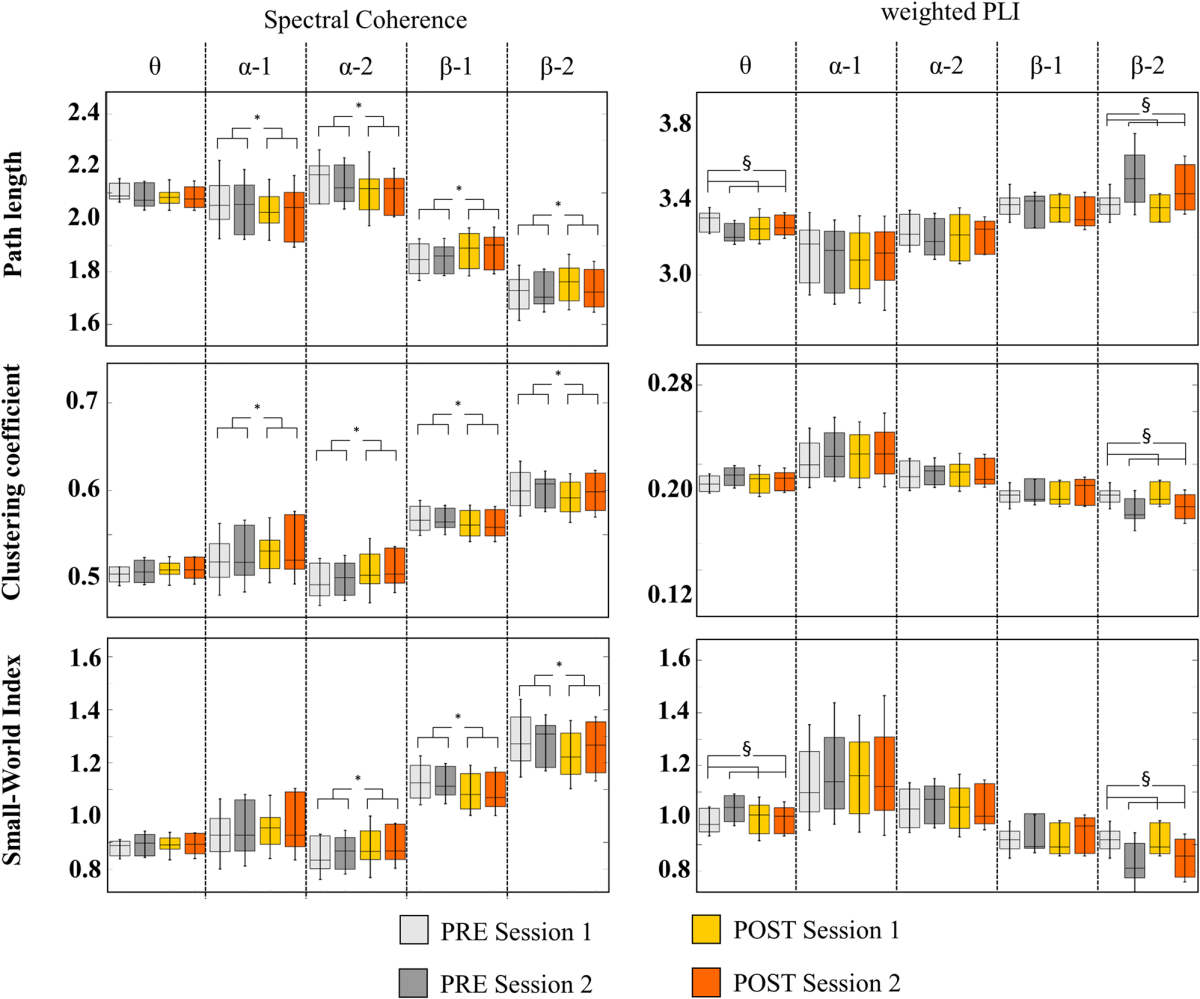
Box plot of graph measures based on Spectral coherence (left column) and weighted PLI (right column) functional connectivity estimation. Measures were obtained from two experimental sessions within 1 week investigating EEG resting states before (PRE) and after (POST) an acute bout of 10 min moderate-intensity running on the treadmill. Boxes display the range of values including group mean and standard deviations for for four conditions: PRE Session I (light grey), PRE Session II (dark grey), POST Session I (yellow) and POST Session II (orange). Graph outcomes analyzed were characteristic path length (upper figure), clustering coefficient (middle figure) and Small-World Index (lower figure). *Significant main effect of exercise (PRE vs. POST) in both sessions (p < 0.05), ^§^significant main effect of session day (Session 1 vs. Session 2) in all pre and post conditions (p < 0.05).

For WPLI-based measures, significant main-effects of session day but not for for exercise were observed. Small-world Index increased significantly in the theta band from Session 1 to Session 2 in both conditions (p = 0.04, F = 4.93), while SWI in the beta-2 band decreased significantly from Session 1 to Session 2 (p = 0.0001, 41.74). Accordingly, PL decreased in the theta band (p = 0.02, F = 6.81). In the beta-2 band, PL (p = 0.0002, F = 26.28) and CC (p = 0.0002, F = 43.68) decreased from Session 1 to Session 2 in both conditions. An overview of graph outcomes is presented in Fig. 1 and statistical results are provided in Table 2.

**Table 2:** Overview of ANOVA results for physiological and EEG outcomes before and after 10 min of running on the treadmill.

	ANOVA					
	Exercise		Session		Interaction	
	p	F	p	F	p	F
Lac (in mmol/l)	0.11	3.01	**0.03**	5.95	**0.01**	8.13
BS	**0.000001**	71.7	**0.005**	11	0.08	3.62
HR (% HR_max_)	**0.000001**	119.35	0.52ss	0.42	0.19	1.90
SWI wPLI θ	0.22	1.58	**0.04**	4.93	0.12	2.82
SWI Coh θ	0.60	0.28	0.11	2.90	0.20	1.80
SWI wPLI α^1^	0.91	0.01	0.10	3.02	0.35	0.92
SWI Coh α^1^	0.07	3.67	0.35	0.93	0.87	0.03
SWI wPLI α^2^	0.96	0.002	0.56	0.34	0.52	0.43
SWI Coh α^2^	**0.006**	10.35	0.32	1.04	0.36	0.89
SWI wPLI β^1^	0.94	0.01	0.19	1.89	0.41	0.72
SWI Coh β^1^	**0.003**	12.48	0.46	0.58	0.62	0.26
SWI wPLI β^2^	0.58	0.31	**0.0001**	41.74	0.65	0.22
SWI Coh β^2^	**0.002**	15.48	0.9	0.02	0.08	3.5
CC wPLI θ	0.81	0.06	0.07	3.81	0.22	1.61
CC Coh θ	0.09	3.37	0.10	3.05	0.20	1.86
CC wPLI α^1^	0.79	0.07	0.06	4.40	0.29	1.22
CC Coh α^1^	**0.02**	6.56	0.40	0.75	0.91	0.01
CC wPLI α^2^	0.51	0.46	0.52	0.45	0.90	0.02
CC Coh α^2^	**0.002**	14.49	0.40	0.76	0.43	0.66
CC wPLI β^1^	0.55	0.38	0.25	1.42	0.36	0.87
CC Coh β^1^	**0.02**	6.61	0.54	0.40	0.61	0.27
CC wPLI β^2^	0.35	0.93	**0.0002**	43.68	0.69	0.16
CC Coh β^2^	**0.01**	9.01	0.83	0.04	0.09	3.44
PL wPLI θ	0.79	0.07	**0.02**	6.81	0.22	1.61
PL Coh θ	0.24	1.54	0.15	2.33	0.27	1.33
PL wPLI α^1^	0.53	0.41	0.25	1.48	0.42	0.69
PL Coh α^1^	**0.04**	5.19	s0.35	0.94	0.66	0.20
PL wPLI α^2^	0.66	0.19	0.55	0.37	0.38	0.80
PL Coh α^2^	**0.006**	10.06	0.19	1.90	0.60	0.28
PL wPLI β^1^	0.44	0.62	0.17	2.00	0.47	0.57
PL Coh β^1^	**0.01**	8.56	0.46	0.59	0.71	0.14
PL wPLI β^2^	0.29	1.22	**0.0002**	26.28	0.50	0.48
PL Coh β^2^	**0.005**	15.47	0.90	0.02	0.08	3.51

The table presents p- and F-values of repeated measures ANOVA with the factors Exercise (PRE vs. POST) and Session (Session I vs. Session II) as well as analyses for Interaction effects.

Significant effects (p < 0.05) are marked as bold. Significant effects are marked as bold. Blood lactate values (Lac) obtained from the right earlobe and Borg Scale (BS) were assessed before (PRE) and immediately after (POST) running on the treadmill. Average resting heart rate (HR_rest_) is determined as the 5-min average heart rate recorded during the EEG resting state measurements performed in a sitting position. Small-World-Index (SWI), clustering coefficient (CC) and characteristic path length (PL) derived from Coherence (Coh) respectively weighted phase-lag-index (wPLI) are based on 5-min resting state EEG. SWI measures are presented for theta (θ, 4–8 Hz), alpha-1 (α^1^, 8–10.5 Hz), alpha-2 (α^2^, 10.5–13 Hz), beta-1 (β^1^, 13–20 Hz) and beta-2 (β^2^, 20–30 Hz) frequency bands.

### Relative reliability of brain graphs

**Figure 2:**
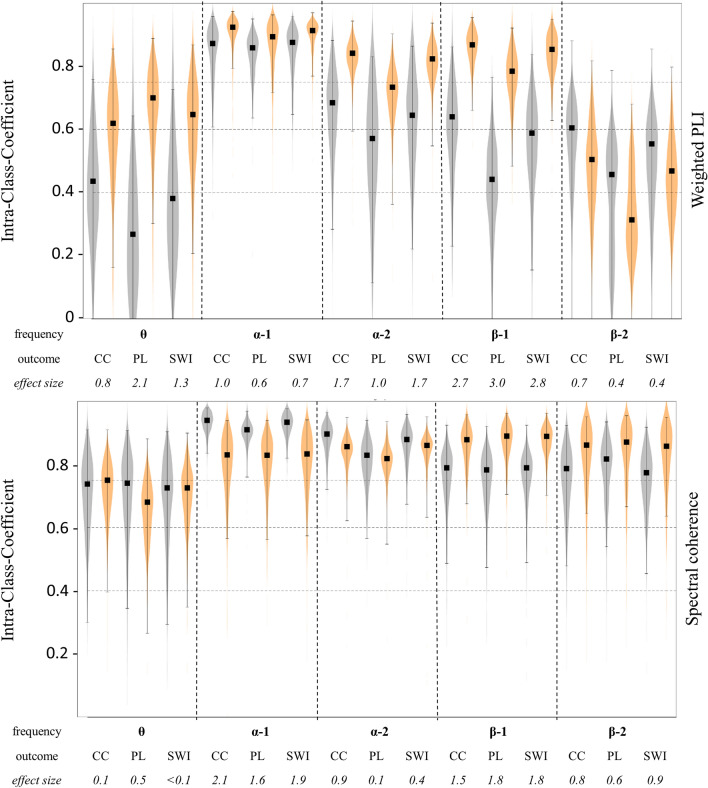
Violin plots of Intraclass-Correlation-Coefficient (ICC) values for graph measures based on weighted PLI (upper graph) and spectral coherence (lower graph) functional connectivity estimation. Measures were obtained from two experimental sessions within one week investigating EEG resting states before (PRE, grey violins) and after (POST, orange violins) an acute bout of 10 min of moderate-intensity running on the treadmill. Violins display the distribution of ICC values based on a resampling of k = 1000 iterations using the *bootes* function (Di Plinio 2020). For each pair of distributions (PRE vs. POST), effect size Cohen’s d was computed based on k ICC values per condition. Black squares indicate the ICC values of the original sample, brackets indicate the 95% confidence intervals. *CC* clustering coefficient, *PL* characteristic path length, *SWI* small-world index. Graph measures are computed for theta (θ, 4 to 8 Hz), alpha-1 (α-1, 8 to 10.5 Hz) and alpha-2 (α-2, 10.5 to 13 Hz), beta-1 (β-1, 13 to 20 Hz) and beta-2 (β-2, 20 to 30 Hz) frequency bands*.* Horizontal lines indicate thresholds for fair (0.4, lower line), good (0.6, midline) and excellent (0.75, upper line) ICC interpretation.

ICC values for relative reliability between graph measures of two sessions within one week ranged from poor to excellent (overall range: 0.27 to 0.94). An overview of the ICCs, including 95% CIs for wPLI and Coh graph measures, is presented in Fig. 2. With regards to the graph outcome analyzed, CC (0.77) and SWI (0.75) revealed excellent mean ICCs, while a good mean ICC was observed for PL (0.71). Further, ICCs observed for Coh-based graph measures were excellent in mean (0.83), while those obtained from wPLI-based networks were good (0.66). Additionally, alpha-1, alpha-2, and beta-1 revealed excellent ICCs (0.88 vs 0.79 vs. 0.77), while theta- and beta-2 based networks revealed good ICC values (0.62 vs. 0.66).

**Figure 3:**
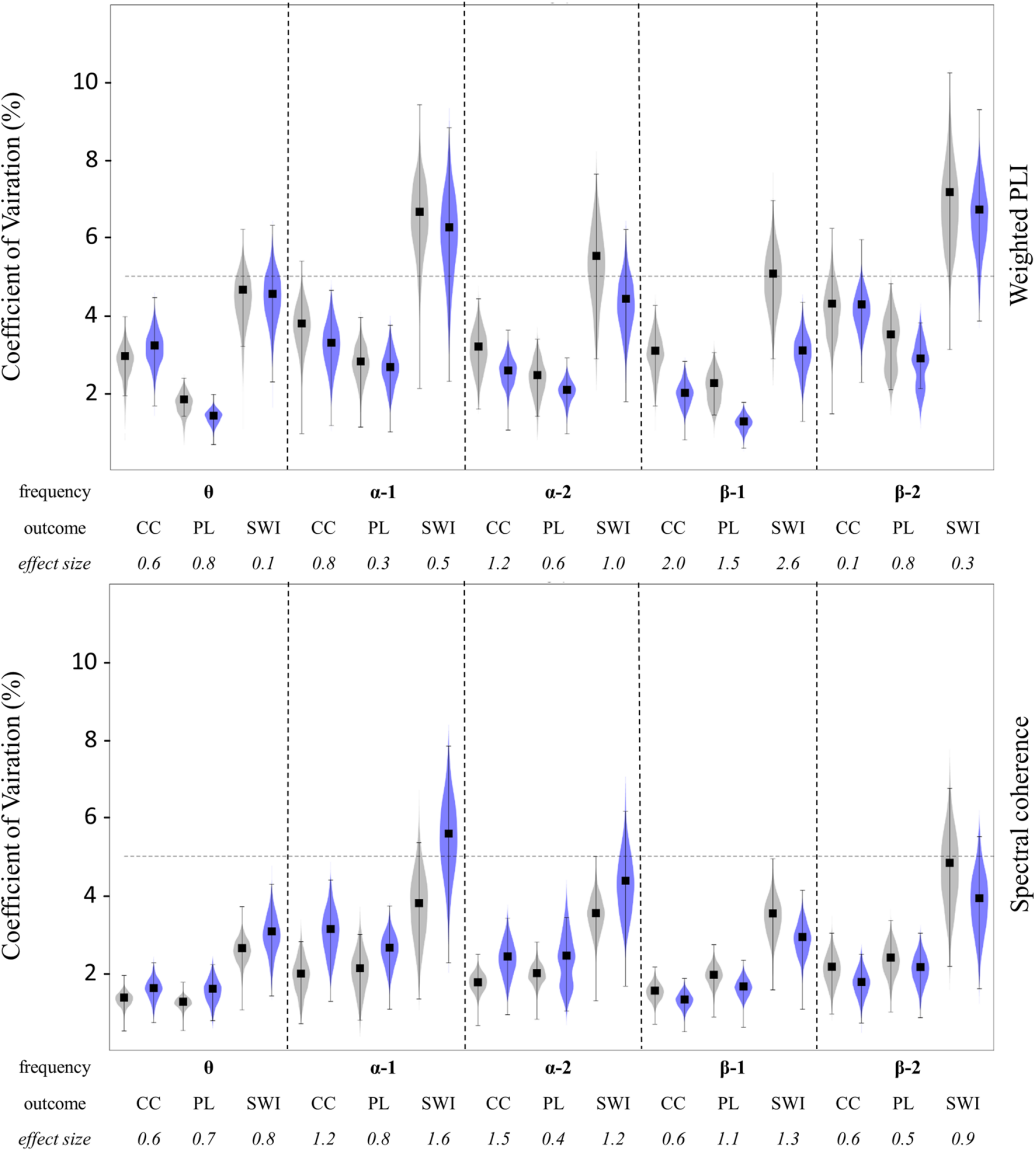
Violin plots of Coefficient of Variation (CoV) values for graph measures based on weighted PLI (upper graph) and spectral coherence (lower graph) functional connectivity estimation. Measures were obtained from two experimental sessions within one week investigating EEG resting states before (PRE, grey violins) and after (POST, purple violins) an acute bout of 10 min of moderate-intensity running on the treadmill. Violins display the distribution of CoV values based on a resampling of k = 1000 iterations using the *bootes* function (Di Plinio 2020). For each pair of distributions (PRE vs. POST), effect size Cohen´s d was computed based on k CoV values per condition. Black squares indicate the CoV values of the original sample, brackets indicate the 95% confidence intervals. *CC* clustering coefficient, *PL* characteristic path length, *SWI* small-world index. Graph measures are computed for theta (θ, 4 to 8 Hz), alpha-1 (α-1, 8 to 10.5 Hz) and alpha-2 (α-2, 10.5 to 13 Hz), beta-1 (β-1, 13 to 20 Hz) and beta-2 (β-2, 20 to 30 Hz) frequency bands. Horizontal line indicates the 5% threshold for CoV interpretation.

To analyze the effect of exercise on the reliability of graph outcomes, a bootstrap analysis on the original data samples was performed. Based on effect size estimation, exercise revealed small to large effect sizes on ICC. Hereoff, ES differed with regards to FC measure, frequency band and outcome (Fig. 1). Overall, wPLI based graph measures indicated small to largely increased ICC values at POST (mean ES: 1.2), which was particularly observed in the alpha-1 (mean ES: 1.5) and theta frequency bands (mean ES: 1.5). For Coh based ICCs, small to large negative effects of exercise on ICC were observed (mean ES: 1.00), which was most prominent in the alpha-1 band (mean ES: 1.9). An overview of ES for SWI measures is provided in Table 3. A visualization of ICC values for all graph outcomes, including 95% confidence intervals and bootstrapped distributions based on a resampling of k = 100 iterations, is presented in Fig. 3.

**Table 3:** Overview of results revealed from reliability analysis for small-world-index values before and after 10 min of running on the treadmill.

		Descriptives		Relative reliability			Absolute reliability		
		Session 1	Session 2	ICC	[LB UB ]	ES	CoV	[LB UB]	ES
θ	wPLI_REST_	0.92 ± 0.05	0.96 ± 0.06	0.38	[0.0 0.73]		4.65	[6.12 3.09]	
	wPLI_POST_	0.94 ± 0.08	0.94 ± 0.06	0.65	[0.21 0.87]	1.30	4.55	[6.83 2.79]	0.10
	Coh_REST_	0.99 ± 0.04	1.01 ± 0.06	0.73	[0.29 0.90]		2.63	[4.22 1.56]	
	Coh_POST_	0.98 ± 0.06	0.98 ± 0.06	0.73	[0.35 0.90]	0.06	3.07	[4.73 1.86]	0.91
α^1^	wPLI_REST_	1.08 ± 0.20	1.13 ± 0.22	0.88	[0.65 0.96]		6.66	[11.21 3.89]	
	wPLI_POST_	1.10 ± 0.22	1.13 ± 0.26	0.91	[0.77 0.97]	0.85	6.26	[10.23 3.70]	0.39
	Coh_REST_	1.05 ± 0.15	1.07 ± 0.16	0.93	[0.82 0.98]		3.79	[6.25 2.23]	
	Coh_POST_	1.06 ± 0.14	1.07 ± 0.15	0.83	[0.57 0.94]	1.83	5.58	[8.92 3.33]	1.56
α^2^	wPLI_REST_	0.97 ± 0.09	0.99 ± 0.09	0.64	[0.22 0.86]		5.52	[8.17 3.41]	
	wPLI_POST_	0.98 ± 0.12	0.98 ± 0.09	0.82	[0.55 0.94]	1.53	4.42	[7.06 2.63]	1.09
	Coh_REST_	0.95 ± 0.10	0.97 ± 0.10	0.88	[0.67 0.96]		3.54	[5.79 2.09]	
	Coh_POST_	0.97 ± 0.13	0.98 ± 0.10	0.86	[0.63 0.95]	0.37	4.37	[7.09 2.59]	1.27
β^1^	wPLI_REST_	0.92 ± .07	0.94 ± .08	0.59	[0.15 0.84]		5.07	[7.27 3.19]	
	wPLI_POST_	0.92 ± .07	0.94 ± .08	0.85	[0.63 0.95]	2.77	3.09	[4.92 1.85]	2.53
	Coh_REST_	1.14 ± 0.10	1.12 ± 0.10	0.79	[0.49 0.92]		3.53	[5.50 2.12]	
	Coh_POST_	1.10 ± 0.10	1.09 ± 0.10	0.89	[0.70 0.96]	1.77	2.92	[4.78 1.73]	1.10
β^2^	wPLI_REST_	0.92 ± 0.07	0.83 ± 0.11	0.56	[0.00 0.85]		7.17	[11.24 4.10]	
	wPLI_POST_	0.92 ± 0.07	0.85 ± 0.09	0.47	[0.00 0.80]	0.42	6.72	[9.60 4.15]	0.43
	Coh_REST_	1.29 ± 0.15	1.28 ± 0.11	0.77	[0.45 0.92]		4.83	[7.02 3.18]	
	Coh_POST_	1.23 ± 0.13	1.25 ± 0.13	0.86	[0.64 0.95]	0.90	3.92	[5.82 2.56]	0.94

Data are presented as outcome means ± standard deviations. For purposes of reliability, table provides Intraclass-Correlation-Coefficients (ICC) as well as Coefficient of Variation (CoV) including lower (LB) and upper bound (UB) of the 95% confidence interval of two sessions performed within one week. Effect size estimations (ES) are based on bootstrap analysis with k = 100 cycles running the bootes function (Di Plinio 2020).

### Absolute reliability of brain graphs

Absolute reliability was analyzed by taking into account the SEM´s proportion with regards to the outcome measure. We observed excellent CoV (in %) for most of the outcomes assessed. Coh-based measures (mean: 2.6%) and wPLI-based measures (mean: 3.7%) both revealed good CoV values. With regards to the graph measures analyzed, SWI (mean: 4.6%), PL (mean: 2.2%) and CC (mean: 2.6%) revealed good average CoV. With regards to the frequency bands of interest, networks metrics derived from theta (2.5%), alpha-1 (3.7%), alpha-2 (3.1%), beta-1 (2.5%) and beta-2 (3.8%) demonstrated good CoV. SWI exceeded the arbitrary threshold of 5% for the following cases: wPLI-Pre-alpha-1 (6.7%) and Post-alpha-1 (6.3%), Coh-Post-alpha-1 (5.6%), wPLI-Pre-alpha-2 (5.5%) and wPLI-Pre-beta-1 (5.1%), WPLI-Pre-beta-2 (7.2%) and WPLI-Post-beta-2 (6.7%).

Bootstrap analysis to detect significant changes of CoV from PRE to POST revealed moderate effect sizes on both Coh (mean ES: 0.91) and wPLI (mean ES: 0.89) derived graph outcomes. An overview of effect sizes for SWI is provided in Table 3. A visualization of CoV values of all graph outcomes, including 95% confidence interval and bootstrapped distribution based on a resampling of k = 100 iterations, is presented in Fig. 3.

## Discussion

The present study aimed to investigate the reliability of brain graphs derived from an uncontrained EEG resting states as well as a resting state after an acute bout of low intensity exercise on the treadmill in male participants, and we expected preceding exercise to modulate graph reliability. The key finding was that the extraction of brain graphs from EEG resting state after low intensity exercise reveals good to excellent relative reliability expressed by ICC for both Coh and wPLI, while brain graphs extracted from unconstrained resting states revealed poor to excellent reliability, with limited reliability especially for wPLI based graphs. Bootstrap analysis revealed a beneficial effect of exercise on wPLI-based ICC values, while exercise reduced ICC derived from Coh based measures particularly in the alpha-1 frequency band. Moreover, CoV demonstrated good absolute reliability before and after exercise. The present findings demonstrate that resting state brain graphs obtained after exercise may represent a reliable measure to analyze brain network efficiency in the field of exercise science.

The novel approach of this study was to analyze the reliability of brain graphs during resting state after physical activity and to compare that with the corresponding unrestricted resting state brain graphs before exercise. Specifically, we observed that preceding exercise increases the relative reliability of wPLI resting state graph measures to good-to-excellent as compared to poor-to-fair reliability in an unconstrained situation. Somehow contradictory, the reliability of Coh based graph measures decreased but remained good to excellent after exercise. Similar modulations of reliability were observed for absolute reliability metrics, with CoV values being slightly increased for Coh-based measures but decreased for wPLI based measures. Overall absolute reliability of graph measures remained mainly below 5%, which is interpreted as a good value in exercise science contexts of repeated measures^23^. The different modulations of brain graph reliability depending on FC estimator appear reasonable when the nature of the respective FC estimation method is considered and are in line with previous network analysis studies observing differences between Coh and PLI networks in different populations^24,25^. Whereas the wPLI detects non-zero phase lags and is less prone to spurious connections based on volume conduction and common sources, Coh-based functional networks rely on signal amplitude and volume conduction and therefore even resemble zero-phase lags in the signal^26^. Since systematic exercise-induced modulations of cortical activity are commonly expressed by changes in spectral power and signal amplitude^1^, this effect may contribute to changes in functional connectivity estimated by amplitude-sensitive FC measures. In contrast, wPLI metrics are suggested to be more sensitive in detecting true non-zero phase synchronization indepedendent from changes in amplitude and power and eliminate zero-phase contributions^26^. Accordingly, it remains open whether an acute bout of low-to-moderate exercise is sufficient to induce systematic changes in global brain network characteristics or whether amplitude and power changes of single neural ensembles contribute to observed findings in Coh-based networks due to volume conduction. According to previous investigations, more intense bouts might be needed to modulate brain network characteristics estimated by non-zero lag correlations on a global level^9,17^. However, the overall reliability analysis demonstrates good to excellent overall reliability, for both wPLI and Coh networks, especially after exercise. This information is from high relevance for future studies when interpreting intra-individual changes based on the expected intra-individual variations of such measures^23^.

A possible mechanism subserving the phenomenon of modulated resting state reliability after exercise may be attributed to the potential neuro-regulating effect of physical exercise^2^. In the present study, several Coh-based brain network metrics (alpha-2, beta-1 and beta-2) revealed significant differences from pre to post in line with increases in resting heart rate and subjective rating of perceived exertion. Since acute bouts of exercise induce the release of several neurochemicals, such as lactate, cortisol and brain-derived neurotrophic factor, the neurochemical milieu of the central nervous system may change after exercise^2^. In particular, even short bursts of exercise were shown to upregulate neurotransmitter release like reported for gamma-aminobutyric-acid^27^ and therefore modulate neural processing and functional brain connections. Moreover, it is suggested that sustained endurance activities such as running or cycling modulates the afferent feedback to central and parietal brain areas and affect brain activity up to 30 min after exercise cessation^7^. As the central nervous system remains modulated after exercise, the activation of functional pathways due to exercise might be suggested to modulate brain network reliability. While several Coh-based metrics were modulated by acute exercise, no significant changes from PRE to POST were observed for wPLI-based brain network metrics. It might be assumed that the short and moderate intensity exercise bout in the present study was not sufficient to systematically up- or downregulate small-world characteristics based on non-zero phase lag estimation across participants. However, increased reliability may reveal that a short bout of low-to-moderate exercise may function as a reset of the central nervous system towards a more reliable state. In particular, the exercise-induced release of neurotrophics may bidirectionally regulate the state of the central nervous system depending on the participants state and trait by upregulating positive affect and reducing negative affect^28^. Therefore, our overall findings agree with previous investigations reporting that functional brain networks are more stable when individuals are involved into a task or context compared to unconstrained conditions^21,29^. To elucidate the modulating effect of exercise on brain network metrics, further investigations are required that analyze exercise intensity and exercise duration. Nevertheless, these studies should consider incorporating preceding low-to-moderate-intensity activity in addition to the resting baseline for a more reliable reference to investigate exercise-induced changes in brain networks.

In the present study, ICCs for global graph metrics ranged from poor to excellent. Previous studies predominantly reported slightly lower ICCs for graph-based brain network measures which majorly ranged from poor too good. While Kuntzelmann et al. reported poor to good ICCs depending on the selected FC measure (either wPLI or Coh), Hardmeier et al. reported fair to good ICCs for wPLI-based graph measures in different frequency bands. A possible reason for the relatively high number of excellent ICC values observed in our study compared to previous graph reliability studies, in particular in the alpha-1 and alpha-2 band, might be related to the increased length and number of epochs chosen. On the one hand, it was suggested that the analysis of longer epochs incorporates more cycles per frequency for FC analysis and results in more variable data^19^. Further, Marquetand et al. reported systematically increasing FC reliability with increased numbers of data epochs due to reduced impact of noise and spurious oscillations on brain connectivity. Accordingly, the higher variance in our data might have reduced the risk of overestimating edges between nodes based on spurious oscillations or noise, resulting in increased reliability of networks compared to previous studies^30^. Further taking into account the observed modulating effect of preceding exercise on brain graph reliability, global graph measures obtained from sufficient data points after exercise reveal a reliable measure for the analysis of resting state brain networks.

This is the first study to analyze absolute reliability of graph measures, which is particularly relevant for the interpretation of longitudinal modulations of graph measures, since intra-individual variations can reveal a threshold of statistical meaningfulness for observed measurement-to-measurement changes (Lohse et al.). In the present study, we mainly observed good CoVs for graph measures obtained before and after exercise, ranging from 1.25 to 7.17%. It can consequently be assumed that repeated analysis of graph measures do not underly strong absolute intra-individual changes^31^. This observation is in line with observations in elderly^32^ and infant populations^33^, where repeated assessments of brain graphs within days did not obtain significant differences from test to test. Therefore, graph measures not only display adequate relative, but also absolute reliability and could be feasible in displaying intra-individual adaptations to exercise in repeated measures designs.

### Methodological considerations

Although our findings demonstrated good reliability for post-exercise resting state graph measures, some limitations of the present study need to be mentioned. Primarily, it needs to be mentioned that our findings are based on sensor-level data, so that possible effects of volume conduction on the computed connectomes, especially for those based on coherence, need to be considered. Further, previous investigations report that connectivity outcomes from source-space in part correlate poorly sensor-level connectivity metrics^34^, so that the present findings should be transferred with caution to source-space approaches. However, since source-space analysis incorporates several additional processing steps, graph reliability after exercise might even differ to a considerable degree within the source-space domain^35^. With regards to the transferability of the present findings, the factor of sex should also be taken into account, since scross-sectional fundings suggest gender- and hormone-based differences in resting state brain networks comparing male and female individuals^36,37^.

Further, the cap was placed on the participants’ head prior to each EEG resting state assessment, so that the participants were not restricted by the cap during exercise. The replacement of the cap may have resulted in systematic error in electrode position from measurement to measurement, which can affect brain network analysis outcomes and consequently test–retest reliability when these deviations are > 0.5 cm^38^. Since digitizing electrode positions requires a minimum of additional time of about 15 min^39^, this procedure was not feasible for the present study analyzing the effects of acute exercise bouts on brain outcomes, but should be considered for future longitudinal studies on brain networks.

The major concern when interpreting the present findings should be assigned to the trade-off between reliability and sensitivity of brain networks^19^. As a brain graph is a result of both functional and structural connections which contribute to an existing connectome, both chronic and acute neuroplastic changes contribute to modulations of brain graphs. While structural connections are expected to remain stable over time, specific functional connections are more likely to rather reflect state than trait and therefore fluctuate more within smaller time periods^40,41^. Even if the analysis of brain graphs is majorly applied to distinguish healthy from diseased populations exposed to long-term neuroplastic changes^42^, first studies have shown that even acute circumstances like fatigue or vigilance correlate with modulations of functional graph outcomes^43,44^. Therefore, brain graphs might be treated as a mixture of long-term, mid-term and short-term modulated brain connectivity which also require for a complex and multimodal interpretation. In line with the above mentioned observations in the existing literature, we observed changes in small-worldedness from Session I to Session II in wPLI-based networks accompanied by reduced lactate and borg scale responses to exercise in the second session. Thus, even in well controlled week-to-week investigations, intra-individual changes like familiarization, experimental anxiety or even subjective well-being should be considered as inherent sources of variability in functional connectivity analysis. To apply brain graphs as possible markers of network efficiency, future studies need to identify outcomes associated with intra-individual changes in the connectome to better understand the its role in sports performance. Furthermore, methodological choices like FC estimation need to be considered as influences on the observed brain network findings^15,19^, which is further supported by the diverging findings in the present study comparing Coh and wPLI.

## Conclusions

The present study indicates that brain graphs are a reliable tool to analyze the modulation of brain networks due to exercise. Both the relative and absolute reliability demonstrated good to excellent values independent from the FC estimator after exercise. Due to the good reliability, chosen graph measures enable not only the investigation of acute, but also longitudinal studies in exercise-scientific contexts. Accordingly, graph theory possibly provides a complementary perspective on acute and chronic neuroplastic changes of brain function associated with exercise compared to the established activity analysis^1^. However, upcoming studies need to investigate long-term reliability and variability of extracted brain networks to display chronic changes within brain network efficiency induced by exercise. Due to the advantages of mobile EEG, graph applications may allow us to monitor (neuro-) physiological responses to exercise in field settings with modifications of different contextual variables like population group, gender, training status, modality, intensity, frequency or volume.

## Methods

### Participants

**Figure 4:**
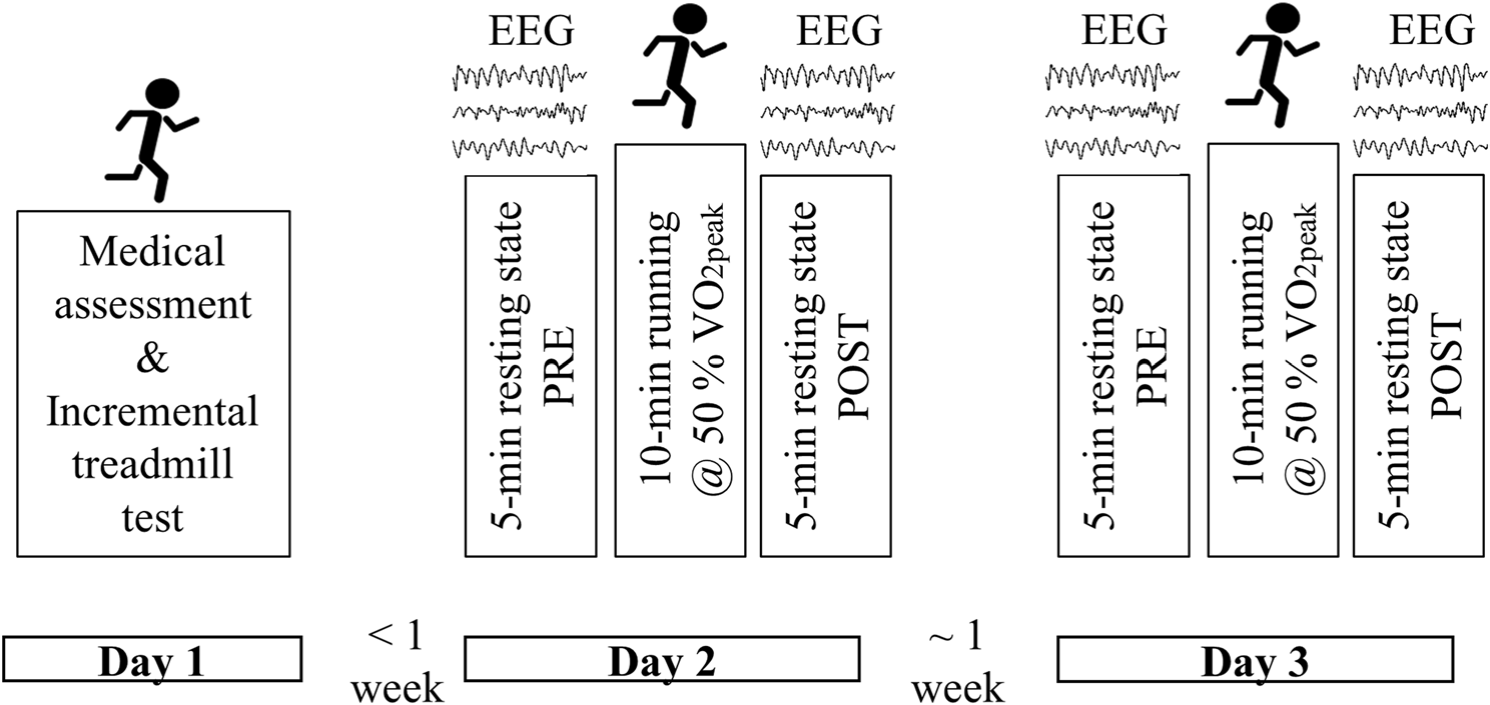
Overview of the experimental procedure to investigate test–retest reliability of graph measures in a resting situation after exercise. Day 1 consisted of a medical assessment and an incremental treadmill test to assess peak oxygen uptake (VO_2peak_). On day 2 and 3, electroencephalography (EEG) resting state was assessed before (PRE) and after (POST) running for 10 min at the speed corresponding to 50% of individual VO_2peak_.

Fifteen healthy male participants (age: 24.5 ± 3.4 years, body height: 182.6 ± 8.8 cm, body mass: 75.4 ± 9.9 kg) who performed exercise at least three times (exercise time/week: 8.0 ± 3.7 h; endurance exercise time/week: 5.2 ± 4.5 h) each week took part at the present study. The present study included 3 days of testing: On day 1, each participant performed a medical assessment including a 12-lead resting electrocardiogram (custo cardio 100 BT, customed, Ottobrunn, Germany) which was screened and approved by a physician. Further, all participants performed an aerobic assessment test to detect peak oxygen uptake (VO_2peak_) and the corresponding peak running speed (vVO_2peak,_ mean: 51.0 ± 5.2 ml/min/kg) during an incremental ramp protocol. Based on the individual VO_2peak_, exercise load was defined for the consecutive trials on day 2 and day 3. The two sessions including EEG recording (day 2 and day 3) were separated by ~ 1 week. Both sessions for each participant were scheduled at the same time of the day to control for possible effects of circadian rhythm on resting state EEG analysis. An overview of the experimental procedure is presented in Fig. 4. Written informed consent was obtained from each participant and the study was approved by the local research and ethics committee of Paderborn University. All procedures of the present study were conducted in accordance with the Declaration of Helsinki.

### Procedures

For each of the two EEG sessions (day 2 and day 3), participants ran through the same standardized protocol. After arriving at the lab, an EEG cap with 64 passive wet-electrodes (RNET, BrainProducts, Gilching, Germany) was fitted to the participants head and connected to a wireless Bluetooth amplifier (LiveAmp, BrainProducts, Gilching, Germany). The impedance level of the electrodes was reduced to 50 kOhm, FCz was chosen as the reference and the sampling rate was set at 500 Hz. Each resting state EEG assessment lasted for 5 min. For the unrestricted resting situation (PRE), participants were asked to sit on a comfortable chair in front of a white wall and keep their eyes open. After the PRE period, the EEG cap was removed, and participants were asked to run for 10 min at an individually determined speed corresponding to 50% vVO_2peak_. Based on previous analysis of the protocol (Büchel et al.), we decided to employ a moderate rather than a high endurance exercise intensity. While high intensity exercise is associated with both negative and positive effects on brain function and cognitive variables, moderate intensity induces beneficial changes in brain function, cognition and mood^2,45,46^. After 10 min running, participants sat down on the chair again and the EEG cap was applied a second time according to the international 10–20 system and aligned with regards to the anatomical landmarks of nasion, inion, and left and right preauricular. Due to the quick application of wet electrodes, EEG resting state assessment started within 4 min after running termination (POST). Before PRE and POST, blood lactate was obtained from the right earlobe and the rate of perceived exertion was assessed using the Borg-scale (BS). During the resting state, the heart rate was monitored using a chest belt connected to an ECG sensor (Polar H10, Polar Electronics, Kempele, Finland).

### Graph analysis

For the data processing, the EEG raw data was imported into the EEGLAB toolbox^47^ for MATLAB (Version R2020a, Mathworks Inc., Natick, United States). The EEG signals were processed using a custom-built automated code including a Cleanline filter^48^, a finite impulse response filter between 3 and 40 Hz, the restoration of the reference-electrode FCz, re-referencing to common average, as well as a down-sampling to 256 Hz. In order to remove non-stereotype artifacts, the clean_rawdata EEGLAB plugin^49^ was applied, which performs automated subspace reconstruction (ASR), a component-based method to effectively interpolate transient or large-amplitude artifacts^50^. After the application of ASR, an adaptive mixture independent component analysis (AMICA, Palmer^51^) was applied to decompose the signal into brain and non-brain signals. The factor *n* (samples/number of channel^2^) = 18.17 was expected to be sufficient for valid AMICA decomposition due to the low amount of artefacts and stationarity of the neural sources^52^. Independent Components (ICs) were labeled applying the ICLabel plugin^53^. Based on visual inspection of scalp map topographies, time course, and frequency characteristics, ICs not labeled as brain resulting from muscle activity, eye movement, ECG, sweat or channel noise were removed from the dataset, in case they did not contain a reasonable contribution of brain oscillation activity. During this step, 8.3 ± 2.6 ICs were removed per dataset (PRE_Session 1_: 8.0 ± 2.7; POST_Session 1_:10.3 ± 2.9; PRE_Session 1_: 7.3 ± 1.8; POST_Session 2_: 7.5 ± 1.8) and allowed to increase the signal-to-noise ratio for further channel-space analysis. Since previous investigations stated that epochs shorter than 4 s possibly overestimate FC measures due to a lack of variance in the data^30,34^, the pruned data was epoched into 8 s windows. Since the number of epochs is further described to affect resting state reliability^20^, we chose epochs overlapping for 4 s to double the amount of epochs for the given number of samples based on real and temporally related data. Further, Allen et al. described that overlapping windows avoid that the position of a given sample in the extracted epoch afftects analysis outcomes^54^. Due to the chosen approach, each datapoint was analyzed twice. Since no study reported the impact of resting states longer than 4 min durations on graph reliability^19^, we limited our analysis to the first 50 epochs of each EEG resting state recording which corresponds to 3.4 min of consecutive EEG data.

To compute brain graphs, the epoched data was imported to the BrainWave software version 0.9.151.7.2^55^. Functional connectivity between EEG channels was derived by means of (i) the wPLI and (ii) Coh. While Coh is a measure of signal covariance, taking into account signal phase and signal amplitude of two time-series, the wPLI is an index of the asymmetry in the distribution of phase differences calculated only on the base of instantaneous phases of two time-series. Both methods reveal measures ranging from 0 to 1, while 1 represents the highest degree of functional connectivity^18^. While phase-lag measures are reported to be less sensitive to signal amplitudes^56^ and might therefore be less prone to exercise-induced changes of signal amplitudes and volume conduction effects^3^, Coh has shown to be more reliable when analyzing slow frequency bands like theta (5–8 Hz, Kuntzelman and Miskovic). Therefore, both Coh and wPLI were computed on the scalp-level for each possible connection between two electrodes for each epoch, resulting in fifty 65-by-65 grids for each participant and each condition per FC measure. The FC computation was performed on band-pass filtered data in the previously defined frequency bands of interest theta (5–8 Hz), alpha-1 (8–10.5 Hz) and alpha-2 (10.5 to 13 Hz), beta-1 (13 to 20 Hz) and beta-2 (20 to 30 Hz). The delta and lower gamme band were not taken into account, as these are prone to artefacts induced by eye^57^ respectively muscle activity^58^ and might be further affected by the bandpass filter chosen. For graph analysis, the matrices were imported to the MATLAB based Brain Connectivity Toolbox^59^. Based on the FC grids, weighted undirected graphs were computed as they are suggested to give more specific information on the degree of connectivity between two nodes than binarized values. To computed weighted brain graph outcomes, connectivity matrices were then normalized by bounding all FC values to the range from 0 to 1 based on the rank of the maximal and minimum FC value per participant. This measured allowed to obtain comparability despite of the considerable differences of absolute FC measures across participants^60,61^. As such, the weighted network approach allowed to keep all possible connections within the brain network since we did not apply an arbitrary threshold for the removal of weakly connected nodes. For each FC measure and condition, the graph measures CC, PL and SWI were computed on the global level. CC and PL were analyzed as global values and represent the mean values of all channels across the scalp, For the computation of SWI, CC and PL were normalized by dividing each individual value by the mean of all other values over all frequency bands and the individual ratio between normalized CC and normalized PL was computed^13^.

### Statistical analysis

All statistical analyses were performed using Matlab Version R2020a (The Mathworks, Inc, Natick, MA). To assess the relative test–retest reliability of the computed graph measures, we computed the ICC comparing the graph outcomes of Session I and Session II. Different statistical models exist to compute ICC, for the present study ICC estimates and their 95% confidence intervals (CIs) were calculated using the MATLAB *ICC* function^62^ based on single ratings, absolute agreement and a 2-way mixed effects model. In the equation below, MSR is the within-participant standard deviation, MSE is the mean square of the error, MSC is the mean square of columns, k is the number of sessions and n is the number of participants^63^.

$$\frac{MSR-MSE}{MSR+\left(k-1\right)MSE+\frac{k}{n}(MSC-MSE)}.$$

ICCs are traditionally interpreted in a range between 0 and 1, where 1 indicates perfect agreement between two measurements. Values smaller than 0 are interpreted as zero values. In accordance with previous studies on brain network reliability, reliability was rated as poor (< 0.4), fair (> 0.4 to < 0.6), good (> 0.6 to < 0.75) or excellent (> 0.75) depending on the corresponding ICC value (Hardmeier et al.). As a measure of absolute reliability, the coefficient of variation (CoV) was calculated in %, estimating the relative impact of the standard error of measurement (SEM) on the observed score. ICC, SEM and CoV are presented as 95% CIs. To analyze whether graph measures obtained from session I differed significantly from those measured during session II, paired t-tests were performed. In addition to t-tests, a novel approach less sensitive to sample size presented by Di Plinio was performed to quantify the differences in the magnitude of correlations across the experimental conditions PRE and POST using the *bootes* function. The function compares correlations between two parameters across two experimental conditions regardless of the input sample size by bootstrapping the orginal sample. It therefore allows more valid assumptions on condition effects on reliability estimates compared to established approaches biased for bigger sample size^64^. In detail, the function performs *k* cycles of resampling the original data sample to *k* samples of equal size. For each resampling step, a correlation value is obtained between the two given input samples, resulting in a distribution of k correlation values. Therefore, the bootes function creates two distributions of ICCs between Session I and Session II for PRE and POST conditions for each outcome in each frequency band which were then transferred into z-values. Based on mean and standard deviation of each distribution, the effect size (ES) Cohen’s d (Lipsey and Wilson) were computed for the difference between correlations of PRE and POST. In the present study, *k* was to 1000 cycles.

Next to EEG data, (psycho-) physiological outcomes obtained at PRE and POST were analyzed with regards to reliability. Therefore, ICC and CoV (in %) for PRE and POST including 95% CI were calculated for mean HR_rest_, BS and Lac. To obtain information on the changes of the EEG data and the physiological state of the participants from PRE to POST, two-way repeated-measures ANOVA was performed to analyze the effect of exercise (PRE vs. POST) and session day (Session 1 vs. Session 2) on (psycho-) physiological outcomes (p < 0.05). All statistical tests were performed using tailored functions in MATLAB.

## Data Availability

The datasets generated during and/or analysed during the current study are available from the corresponding author on reasonable request.

## References

[1] Gramkow MH, Hasselbalch SG, Waldemar G, Frederiksen KS (2020). Resting state EEG in exercise intervention studies: A systematic review of effects and methods. Front. Hum. Neurosci..

[2] Basso JC, Suzuki WA (2017). The effects of acute exercise on mood, cognition, neurophysiology, and neurochemical pathways: A review. Brain Plast..

[3] Crabbe JB, Dishman RK (2004). Brain electrocortical activity during and after exercise: A quantitative synthesis. Psychophysiology.

[4] Park JL, Fairweather MM, Donaldson DI (2015). Making the case for mobile cognition: EEG and sports performance. Neurosci. Biobehav. Rev..

[5] Gwin JT, Gramann K, Makeig S, Ferris DP (2010). Removal of movement artifact from high-density EEG recorded during walking and running. J. Neurophysiol..

[6] Gutmann B (2015). Effects of physical exercise on individual resting state EEG alpha peak frequency. Neural Plast..

[7] Spring JN, Bourdillon N, Barral J (2018). Resting EEG microstates and autonomic heart rate variability do not return to baseline one hour after a submaximal exercise. Front. Neurosci..

[8] Tamburro G, di Fronso S, Robazza C, Bertollo M, Comani S (2020). Modulation of brain functional connectivity and efficiency during an endurance cycling task: A source-level EEG and graph theory approach. Front. Hum. Neurosci..

[9] Porter S, Silverberg ND, Virji-Babul N (2019). Cortical activity and network organization underlying physical and cognitive exertion in active young adult athletes: Implications for concussion. J. Sci. Med. Sport.

[10] Lin M-A (2021). Resistance-induced brain activity changes during cycle ergometer exercises. BMC Sports Sci. Med. Rehabil..

[11] Farahani FV, Karwowski W, Lighthall NR (2019). Application of graph theory for identifying connectivity patterns in human brain networks: A systematic review. Front. Neurosci..

[12] van Straaten ECW, Stam CJ (2013). Structure out of chaos: Functional brain network analysis with EEG, MEG, and functional MRI. Eur. Neuropsychopharmacol..

[13] Vecchio F, Miraglia F, Maria Rossini P (2017). Connectome: Graph theory application in functional brain network architecture. Clin. Neurophysiol. Pract..

[14] Kaminski M, Blinowska KJ (2018). Is graph theoretical analysis a useful tool for quantification of connectivity obtained by means of EEG/MEG techniques?. Front. Neural Circuits.

[15] Sun S (2019). Graph theory analysis of functional connectivity in major depression disorder with high-density resting state EEG data. IEEE Trans. Neural Syst. Rehabil. Eng..

[16] Peraza LR (2018). Electroencephalographic derived network differences in Lewy body dementia compared to Alzheimer’s disease patients. Sci. Rep..

[17] Büchel D, Sandbakk Ø, Baumeister J (2021). Exploring intensity-dependent modulations in EEG resting-state network efficiency induced by exercise. Eur. J. Appl. Physiol..

[18] Hardmeier M (2014). Reproducibility of functional connectivity and graph measures based on the phase lag index (PLI) and weighted phase lag index (wPLI) derived from high resolution EEG. PLoS ONE.

[19] Kuntzelman K, Miskovic V (2017). Reliability of graph metrics derived from resting-state human EEG. Psychophysiology.

[20] Marquetand J (2019). Reliability of magnetoencephalography and high-density electroencephalography resting-state functional connectivity metrics. Brain Connect..

[21] Deuker L (2009). Reproducibility of graph metrics of human brain functional networks. Neuroimage.

[22] Telesford QK (2017). Detection of functional brain network reconfiguration during task-driven cognitive states. Neuroimage.

[23] Hopkins W, Schabort E, Hawley J (2001). Reliability of power in physical performance tests. Sport. Med..

[24] Zhang L (2020). The influence of different EEG references on scalp EEG functional network analysis during hand movement tasks. Front. Hum. Neurosci..

[25] Briels CT (2020). Reproducibility of EEG functional connectivity in Alzheimer’s disease. Alzheimers. Res. Ther..

[26] Vinck M, Oostenveld R, Van Wingerden M, Battaglia F, Pennartz CMA (2011). An improved index of phase-synchronization for electrophysiological data in the presence of volume-conduction, noise and sample-size bias. Neuroimage.

[27] Maddock RJ, Casazza GA, Fernandez DH, Maddock MI (2016). Acute modulation of cortical glutamate and GABA content by physical activity. J. Neurosci..

[28] LePage ML, Crowther JH (2010). The effects of exercise on body satisfaction and affect. Body Image.

[29] Telesford QK (2010). Reproducibility of graph metrics in fMRI networks. Front. Neuroinform..

[30] Fraschini M (2016). The effect of epoch length on estimated EEG functional connectivity and brain network organisation. J. Neural Eng..

[31] Atkinson G, Nevill AM (1998). Statistical methods for assessing measurement error (reliability) in variables relevant to sports medicine. Sport. Med..

[32] Vecchio F (2020). Human brain networks: A graph theoretical analysis of cortical connectivity normative database from EEG data in healthy elderly subjects. GeroScience.

[33] van der Velde B, Haartsen R, Kemner C (2019). Test-retest reliability of EEG network characteristics in infants. Brain Behav..

[34] Lai M, Demuru M, Hillebrand A, Fraschini M (2018). A comparison between scalp- and source-reconstructed EEG networks. Sci. Rep..

[35] Mahjoory K (2017). Consistency of EEG source localization and connectivity estimates. Neuroimage.

[36] Lisofsky N (2015). Hippocampal volume and functional connectivity changes during the female menstrual cycle. Neuroimage.

[37] Hjelmervik H, Hausmann M, Osnes B, Westerhausen R, Specht K (2014). Resting states are resting traits—An fMRI study of sex differences and menstrual cycle effects in resting state cognitive control networks. PLoS ONE.

[38] Taberna GA, Samogin J, Marino M, Mantini D (2021). Detection of resting-state functional connectivity from high-density electroencephalography data: Impact of head modeling strategies. Brain Sci..

[39] Shirazi SY, Huang HJ (2019). More reliable EEG electrode digitizing methods can reduce source estimation uncertainty, but current methods already accurately identify brodmann areas. Front. Neurosci..

[40] Tozzi L, Fleming SL, Taylor ZD, Raterink CD, Williams LM (2020). Test-retest reliability of the human functional connectome over consecutive days: Identifying highly reliable portions and assessing the impact of methodological choices. Netw. Neurosci..

[41] Geerligs L (2015). State and trait components of functional connectivity: Individual differences vary with mental state. J. Neurosci..

[42] Hallett M (2020). Human brain connectivity: Clinical applications for clinical neurophysiology. Clin. Neurophysiol..

[43] Chen J, Wang H, Wang Q, Hua C (2019). Exploring the fatigue affecting electroencephalography based functional brain networks during real driving in young males. Neuropsychologia.

[44] Al-Shargie F (2019). Brain connectivity analysis under semantic vigilance and enhanced mental states. Brain Sci..

[45] McMorris T, Hale BJ (2012). Differential effects of differing intensities of acute exercise on speed and accuracy of cognition: A meta-analytical investigation. Brain Cogn..

[46] McMorris T, Hale BJ, Corbett J, Robertson K, Hodgson CI (2015). Does acute exercise affect the performance of whole-body, psychomotor skills in an inverted-U fashion? A meta-analytic investigation. Physiol. Behav..

[47] Delorme A, Makeig S (2004). EEGLAB: An open source toolbox for analysis of single-trial EEG dynamics. J. Neurosci. Methods.

[48] Mullen, T. *CleanLine.* Version 1.04. http://www.nitrc.org/projects/cleanline (2012).

[49] Miyakoshi, M. & Kothe, C. *clean_rawdata.* Version 2.1. https://github.com/sccn/clean_rawdata/wiki (2019).

[50] Chang CY, Hsu SH, Pion-Tonachini L, Jung TP (2020). Evaluation of artifact subspace reconstruction for automatic artifact components removal in multi-channel EEG recordings. IEEE Trans. Biomed. Eng..

[51] Palmer, J. *AMICA.* Version 1.5.1. https://sccn.ucsd.edu/~jason/amica_web.html (2015).

[52] Särelä J, Vigário R (2004). Overlearning in marginal distribution-based ICA: Analysis and solutions. J. Mach. Learn. Res..

[53] Pion-Tonachini L, Kreutz-Delgado K, Makeig S (2019). ICLabel: An automated electroencephalographic independent component classifier, dataset, and website. Neuroimage.

[54] Allen JJB, Coan JA, Nazarian M (2004). Issues and assumptions on the road from raw signals to metrics of frontal EEG asymmetry in emotion. Biol. Psychol..

[55] Stam, C. J. *BrainWave* Version 0.9.152.12.26. https://home.kpn.nl/stam7883/brainwave.html (2018).

[56] Stam CJ, Nolte G, Daffertshofer A (2007). Phase lag index: Assessment of functional connectivity from multi channel EEG and MEG with diminished bias from common sources. Hum. Brain Mapp..

[57] Hagemann D, Naumann E (2001). The effects of ocular artifacts on (lateralized) broadband power in the EEG. Clin. Neurophysiol..

[58] Whitham EM (2007). Scalp electrical recording during paralysis: Quantitative evidence that EEG frequencies above 20 Hz are contaminated by EMG. Clin. Neurophysiol..

[59] Rubinov M, Sporns O (2010). Complex network measures of brain connectivity: Uses and interpretations. Neuroimage.

[60] Ciccarelli G (2019). Comparison of two-talker attention decoding from EEG with nonlinear neural networks and linear methods. Sci. Rep..

[61] Mehraram R (2020). Weighted network measures reveal differences between dementia types: An EEG study. Hum. Brain Mapp..

[62] Salarian, A. *Intraclass Correlation Coefficient (ICC)* Version 1.3.0.1. MATLAB Central File Exchange. https://www.mathworks.com/matlabcentral/fileexchange/22099-intraclass-correlation-coefficient-icc (2020).

[63] Koo TK, Li MY (2016). A guideline of selecting and reporting intraclass correlation coefficients for reliability research. J. Chiropr. Med..

[64] Di Plinio S (2020). Testing the magnitude of correlations across experimental conditions. PsyArXiv..

