# Author Correction: EEG‑derived brain graphs are reliable measures for exploring exercise‑induced changes in brain networks

**DOI:** 10.1038/s41598-021-01494-x

**Published:** 2021-11-02

**Authors:** Daniel Büchel, Tim Lehmann, Øyvind Sandbakk, Jochen Baumeister

**Affiliations:** 1 Department Sport & Health, Exercise Science & Neuroscience Unit, Paderborn University, Warburger Str. 100, 33098 Paderborn, Germany; 2 Department of Neuromedicine and Movement Science, Centre for Elite Sports Research, Norwegian University of Science and Technology, Trondheim, Norway

Correction to: *Scientific Reports* 10.1038/s41598-021-00371-x, published online 21 October 2021

The original version of this Article contained errors in the spelling of the authors Daniel Büchel, Tim Lehmann, Øyvind Sandbakk & Jochen Baumeister which were incorrectly given as Büchel Daniel, Lehmann Tim, Sandbakk Øyvind & Baumeister Jochen.

The original Article has been corrected.

